# Bile-duct proliferation as an unexpected side-effect after AAV2-LDLR gene transfer to rabbit liver

**DOI:** 10.1038/s41598-019-43459-1

**Published:** 2019-05-06

**Authors:** Elisa Hytönen, Anniina Laurema, Hanna Kankkonen, Atsushi Miyanohara, Vesa Kärjä, Mika Hujo, Nihay Laham-Karam, Seppo Ylä-Herttuala

**Affiliations:** 1 A. I. Virtanen Institute for Molecular Sciences and Department of Medicine, University of Eastern Finland, Neulaniementie 2, FIN-70210 Kuopio, Finland; 2 BioMediTech Institute and Faculty of Medicine and Life Sciences, University of Tampere, Tampere, Finland; 3 Department of Pediatrics, UC San Diego School of Medicine, La Jolla, CA USA; 4 Department of Pathology, University of Eastern Finland, Kuopio, Finland; 5 School of Computing, University of Eastern Finland, 70211 Kuopio, Finland; 6 Heart Center, Kuopio University Hospital, Kuopio, Finland; 7 Gene Therapy Unit, Kuopio University Hospital, FIN-70211 Kuopio, Finland

**Keywords:** Genetic vectors, Genetic vectors, Genetic vectors, Cardiovascular diseases, Cardiovascular diseases

## Abstract

Familial hypercholesterolemia (FH) is an inherited disease of lipoprotein metabolism caused by a defect in the LDL receptor (LDLR) leading to severe hypercholesterolemia, and associated with an increased risk of coronary heart disease and myocardial infarction. We have developed a gene therapy protocol for FH using AAV2, AAV9 and lentiviral vectors and tested safety and efficacy in LDL receptor deficient Watanabe Heritable Hyperlipidemic rabbits. We show that LV-LDLR produced a significant long-lasting decrease in total serum cholesterol whereas AAV9-LDLR resulted only in a transient decrease and AAV2-LDLR failed to reduce serum cholesterol levels. A significant pathological side effect, bile-duct proliferation, was seen in the liver of AAV2-LDLR rabbits associated with an increased expression of Cyr61 matricellular protein. Special attention should be given to liver changes in gene therapy applications when genes affecting cholesterol and lipoprotein metabolism are used for therapy.

## Introduction

Familial hypercholesterolemia (FH) is an inherited disease of lipoprotein metabolism caused by a defect in the LDL-receptor (LDLR), leading to reduced turnover of LDL and significantly increased plasma cholesterol levels. It is associated with an increased risk of premature coronary heart disease, aortic stenosis and myocardial infarction^1,2^. Statins, bile acid sequestrants and plasma apheresis are used for the management of homozygous FH but at best these treatments only delay the onset of cardiovascular complications, and the only curative treatment is orthotopic liver transplantation^3,4^. The poor availability of transplants, technical difficulty of the procedure and life-long immunosuppression necessitate the need to find alternative treatments.

Since the underlying defect in FH is a single gene mutation, genetic correction via the transfer of a functioning LDLR is a logical approach. The feasibility of FH gene therapy has been shown in previous studies^5–7^. Due to the nature of the disease it is apparent that vector systems that express the desired gene for the lifetime of the individual are needed. Both retroviral and lentiviral vectors have been used to treat FH also in large animal models^7,8^ with promising results, and pilot *ex vivo* gene transfer studies in humans have also been done^9,10^.

Adeno-associated viruses (AAV) are non-pathogenic and replication defective, mediate long-term gene expression and are thought to express genes mainly from episomal forms. Different serotypes have varied tissue tropism and while AAV2 has been widely used in many tissues including liver, many of the recently discovered new serotypes such as AAV9 have proven to be more efficient. AAVs have been used in mouse models of FH with correction of hyperlipidemia and even regression of atherosclerosis^11,12^. However, due to differences in size, immune responses, metabolic rate and susceptibility to atherosclerosis between mice and humans, studies in large animal models are needed before proceeding to clinical trials.

Watanabe heritable hyperlipidemic (WHHL) rabbits are hypercholesterolemic due to a 12 nucleotide deletion in the LDLR gene^13^. They demonstrate a similar lipoprotein profile to FH patients and show progression of atherosclerosis similar to that seen in FH patients. Unlike LDLR−/− mice, WHHL rabbits are not null mutants and are thus less likely to develop antibodies against the transduced rabbit LDLR.

We have previously shown that lentivirus (LV) -mediated LDLR gene transfer to WHHL rabbit liver led to a long-term significant decrease in serum cholesterol levels^7^. Here we extended these studies to a full preclinical safety and toxicology study in WHHL rabbits, and also explored the potential of another long-term vector, AAV, in the treatment of FH. We demonstrate that LVs are more efficient than AAV serotype 2 and 9 vectors in treating severe hypercholesterolemia in FH. We also describe a surprising new finding of bile duct proliferation associated with increased Cysteine Rich Angiogenic Inducer 61 (Cyr61) expression in the liver after AAV2-mediated liver-directed gene transfer. To our knowledge, this is the first study to compare these vector systems for the treatment of FH in a preclinical safety, efficacy and toxicology study to select the optimal vector for further clinical development.

## Results

### Cholesterol levels and clinical chemistry

**Figure 1:**
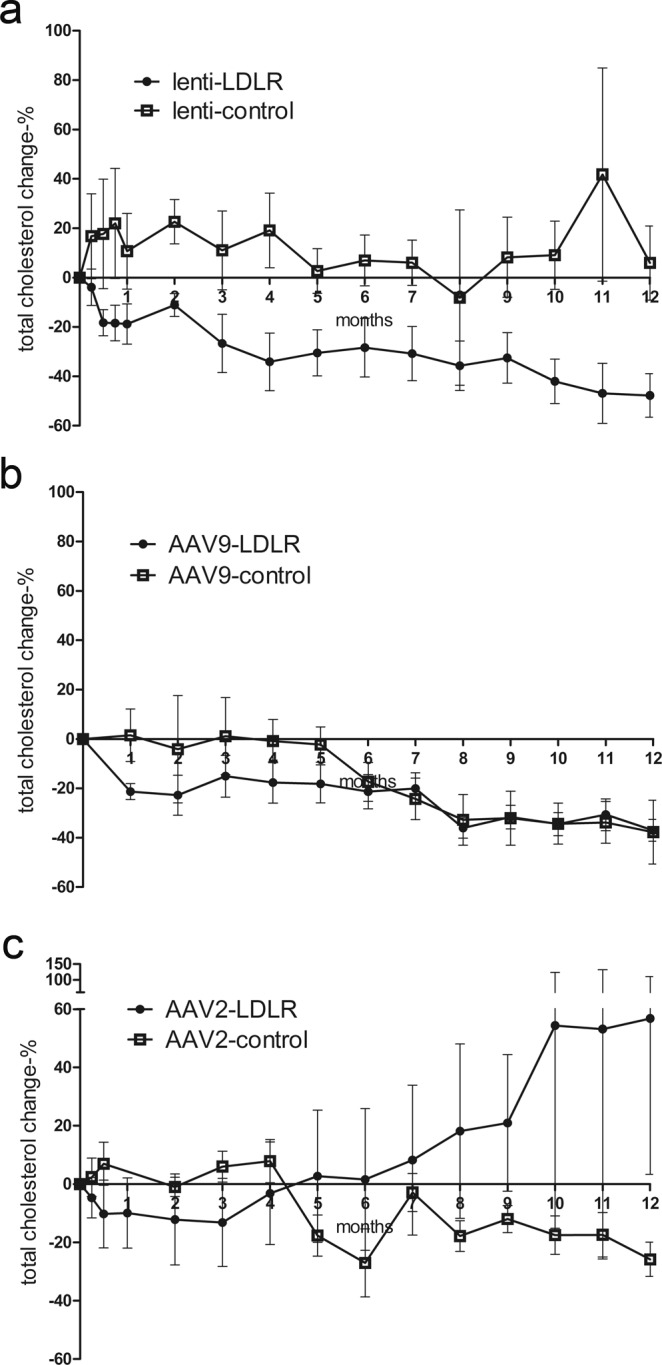
Long-term follow-up of total cholesterol levels. Total cholesterol values were analyzed monthly for the 1-year follow-up after intraportal gene transfer of lenti- and AAV vectors. (**a**) Lenti-LDLR (n = 6) gene transfer led to a significant decrease in total cholesterol compared to lenti-control (n = 4). (**b**) AAV9-LDLR (n = 11) was not significantly more efficient than AAV9-control (n = 6) in lowering serum cholesterol although a trend was seen during the first 6 months whereas AAV2-LDLR gene transfer (n = 5) failed to show a treatment effect at any time point compared to AAV2-control (n = 7) (**c**). Data are expressed as percentage (mean ± SEM) change in comparison to the pre-treatment values in each treated animal. Statistical significance was evaluated using Linear Mixed Models analysis (R statistical software, version 3.2.5). P < 0.05 was considered statistically significant.

Liver-directed intraportal gene transfers of both LV-LDLR and AAV9-LDLR were equally efficient in lowering total cholesterol levels in WHHL rabbits during the first 5 months. In the animals receiving AAV9-LDLR virus 10/11 animals (91%) responded to the gene transfer with a decrease in serum total cholesterol levels 1 month after the gene transfer. Likewise, in the LV-LDLR group 5/6 (83%) animals showed a decrease in total cholesterol during this period (Fig. 1a,b). The average decrease was 21 ± 3% (mean ± SEM) in AAV9-LDLR group compared to an average increase of 2 ± 11% in the AAV9 control group four weeks after gene transfer (Fig. 1b). The decline in total cholesterol levels four weeks after LV-LDLR gene transfer was on average 18 ± 8% and cholesterol values continued to decrease to 47 ± 9% below pre-treatment values one year after gene transfer compared to an increase of 6 ± 15% in LV-control animals (p = 0.033). The total cholesterol level in AAV9 control animals showed a decline starting from 20 weeks after gene transfer and were 38 ± 13% below the pre-treatment values after one-year follow-up compared to a decrease of 37 ± 4% in the AAV9-LDLR group (p = 0.062).

On the other hand, AAV2-LDLR gene transfer did not lead to a reduction in total cholesterol values compared to AAV2-controls at any time point (Fig. 1c). In fact, in some animals from this group the total cholesterol levels increased dramatically during the follow-up despite the LDLR gene transfer. Only one animal in the group responded to LDLR gene transfer with a decrease in total cholesterol for the duration of the follow-up reaching a 65% decrease compared to the pre-treatment value 1 year after the gene transfer (Supplementary Fig. S1). As in the AAV9-control group the total cholesterol levels in the AAV2-control group showed a declining pattern during the follow up (Fig. 1c).

**Figure 2:**
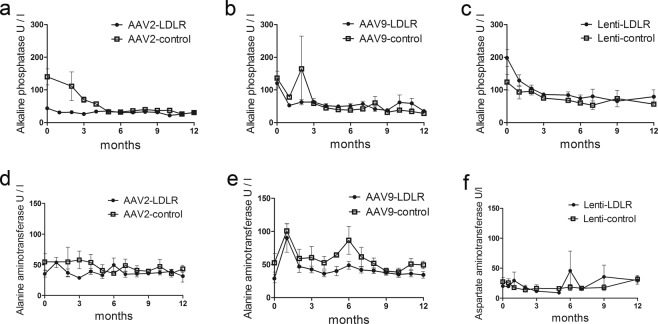
Long-term follow-up of liver enzymes after AAV- and LV-mediated intraportal gene transfers. No significant changes were seen during long-term follow-up of liver enzymes after AAV2-LDLR (n = 5) and AAV2-control (n = 7) (**a,d**), AAV9-LDLR (n = 11) and AAV9-control (n = 6) (**b,e**) or LV-LDLR (n = 6) and LV-control (n = 4) (**c,f**) gene transfers into WHHL rabbit liver. Data is presented as mean ± SEM. Statistical significance was evaluated using Linear Mixed Models analysis (R statistical software, version 3.2.5).

To evaluate safety of the liver directed gene transfer alanine aminotransferase (ALT), alkaline phosphatase (ALP) and aspartate aminotransferase (AST) were analysed from serum samples. There were no significant changes in any of these parameters during the follow-up (Fig. 2a–f).

### Histological findings in the liver

**Figure 3:**
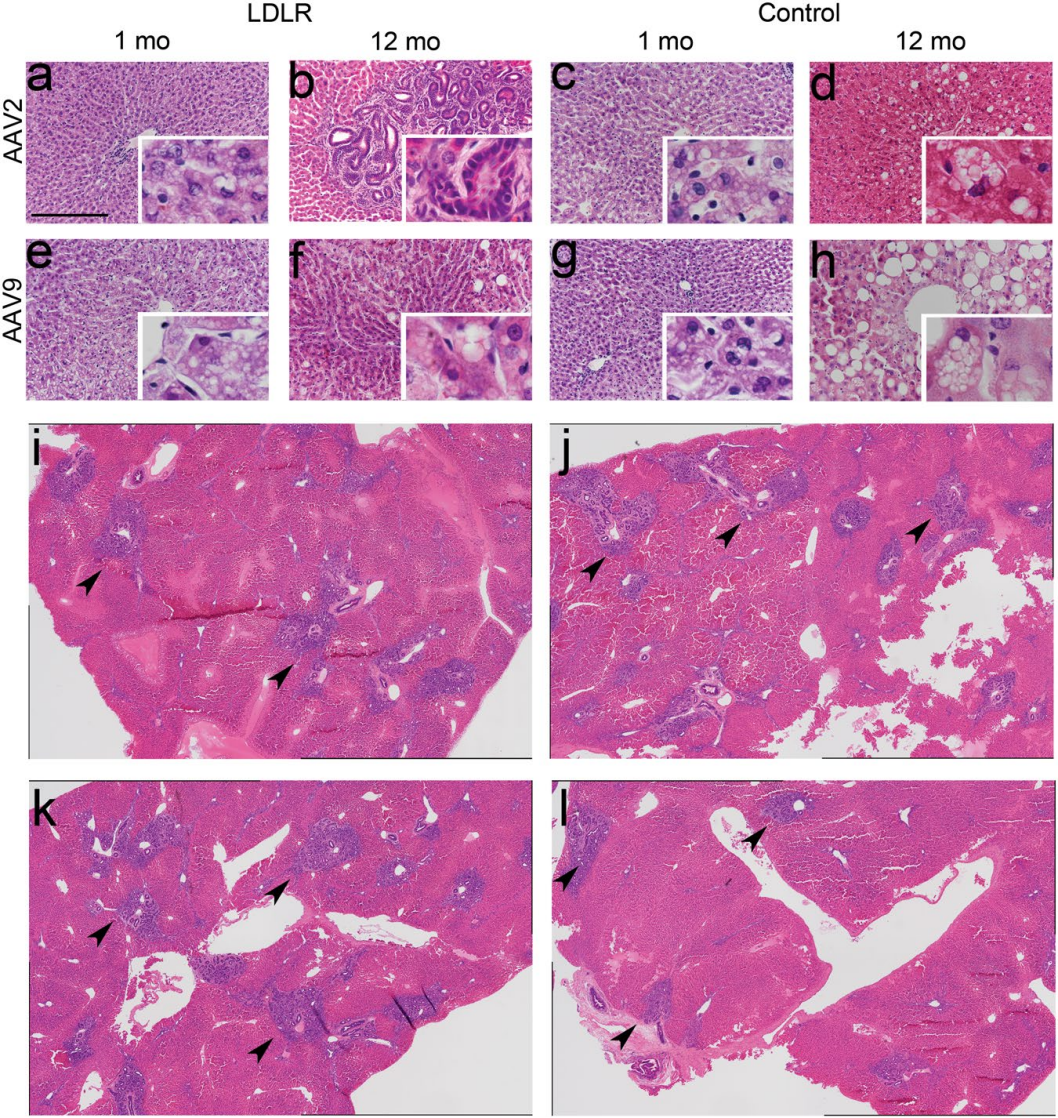
Histological characterization of AAV-control and AAV-LDLR transduced rabbit livers. Representative images of hematoxylin-eosin stained liver sections 1 and 12 months after liver-directed gene transfers. One month after gene transfer ballooning degeneration, microvesicular and macrovesicular steatosis were seen in all AAV-transduced animals (**a,c,e,g**). Widespread bile duct proliferation was seen in AAV2-LDLR transduced animals 12 months after gene transfer (**b**,**i–l**). Magnification 200x, inserts 400x (**a–h**). Scale bar: 100 µm. Composite images comprising four 40x images are shown in (**i–l**) from four different liver lobes from AAV2-LDLR transduced rabbit. Arrowheads indicate generalized pathological findings.

**Figure 4:**
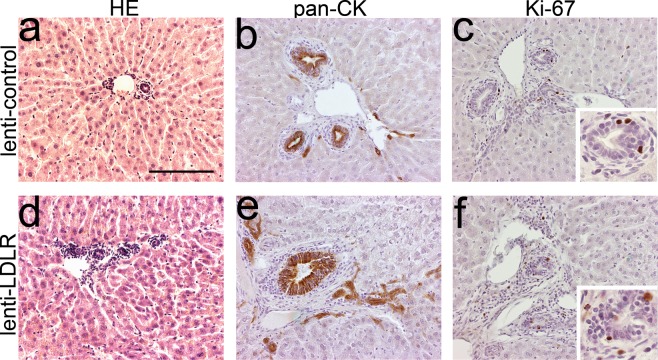
Immunohistochemical characterization of representative rabbit liver samples after gene transfer with lentiviral vectors. Representative images of HE-stained liver 1 year after LV-LDLR (**a**) and LV-control (**d**) gene transfers. (**b**,**e**) Immunohistochemical staining with a pan-cytokeratin antibody stained large and small bile ducts in the liver in both groups. (**c**,**f**) Individual Ki-67 positive cells were seen in bile ducts of LV transduced animals one year after gene transfer. No significant difference was seen between the treatment groups. Magnification 200x, inserts 400x. Scale bar: 100 µm.

**Figure 5:**
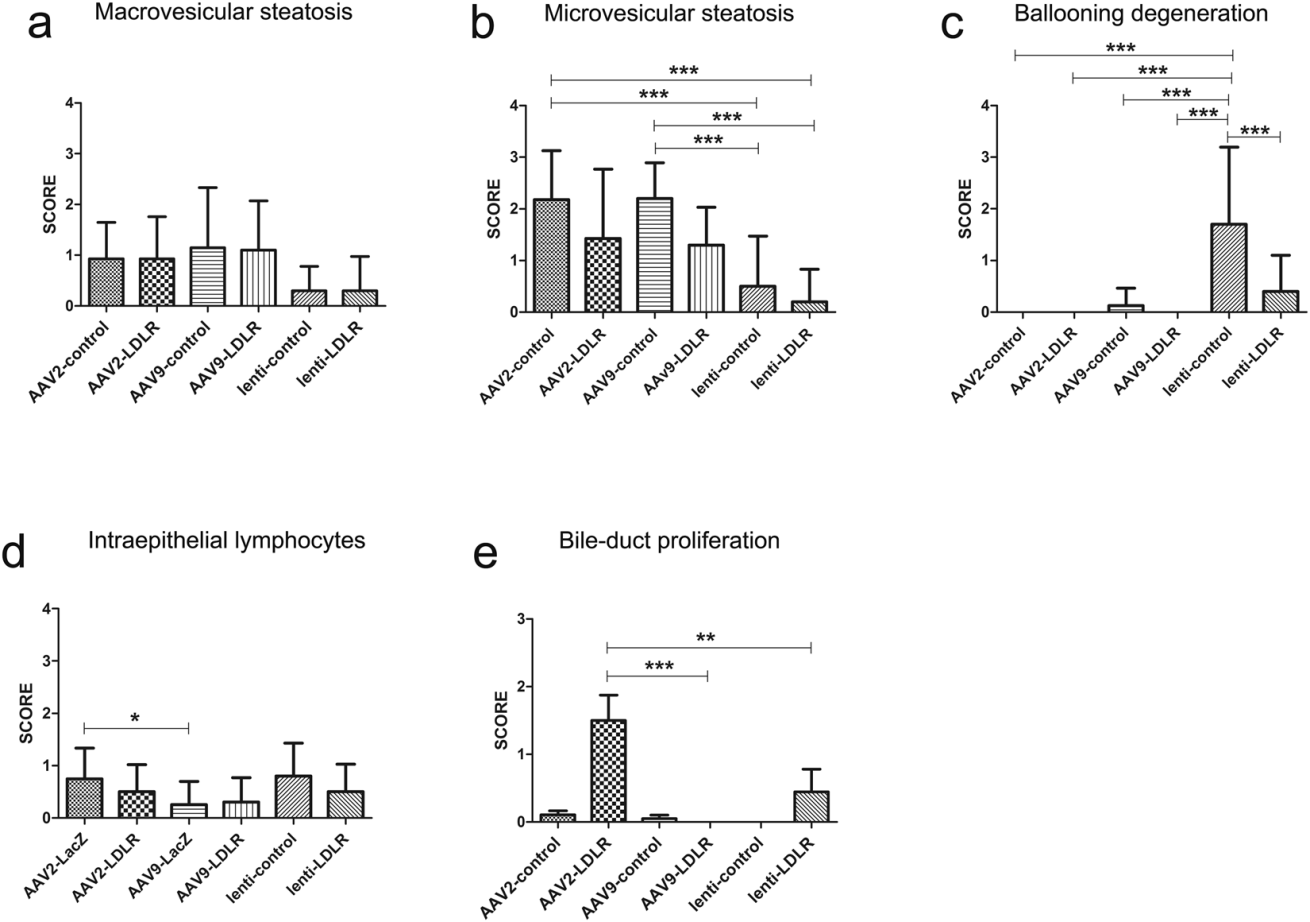
Scoring of histological findings in the liver. HE-stained liver sections were analyzed in a blinded fashion 1 year after AAV2-LDLR (n = 5), AAV2-control (n = 7), AAV9-LDLR (n = 11), AAV9-control (n = 6), LV-LDLR (n = 6) and LV-control (n = 4) gene transfers and changes were scored from 0 to 3 where 0 = no change, 1 = mild change, 2 = moderate and 3 = prominent change. Bars represent mean ± SEM for each treatment group. ***P < 0.0001, **P < 0.001, *P < 0.05, One-way ANOVA with Tukeys multiple comparison test.

Liver sections revealed macrovesicular steatosis in the livers of most animals in all study groups at all time points but there were no statistically significant differences between the groups (Fig. 3a–h). Microvesicular steatosis was also observed in several animals in all groups. There was significantly more microvesicular steatosis in AAV2-and AAV9- control animals one year after gene transfer compared to animals that had received LV-control or LV-LDLR vector (Figs 3a–h, 4a,d and 5a,b). Generally, both macrovesicular- and microvesicular steatosis were more common and more severe in the AAV groups compared to the LV groups (Fig. 5a,b and Supplementary Table S1).

Ballooning degeneration was seen in all AAV-transduced groups at the 1-month time point but could only be detected in 40% of the animals that received AAV9-control vector at the one-year time point (Figs 3a–h and 5c). Severe ballooning degeneration was seen in 60% of the LV-control animals whereas it was seen in 30% of the animals transduced with the LV-LDLR vector at the 1-year time point (Fig. 5c).

Bile duct intraepithelial lymphocytes (IEL) were seen at similar levels in most animals in all study groups although their presence was more prominent at the earlier time points in the AAV2-groups (Fig. 5d, Supplementary Fig. S2 and Supplementary Table S1). The highest amount of IELs was found in the larger bile ducts with lower numbers in the smaller ducts. These cells were identified as T-lymphocytes with immunostaining using an anti-T-lymphocyte antibody (Supplementary Fig. S2). Interphase inflammation was not seen in any of the treatment groups (Supplementary Table S1).

A prominent bile-duct proliferation was seen in the liver, 12 months after AAV2-LDLR gene transfer. Proliferation was seen in all lobes of the liver (Figs 3b,i–l and 5e). These rabbits had also a significant increase in total cholesterol levels during the follow-up (Fig. 1c). The animal with the most severe proliferation was also the one with an increase of ~250% in the total cholesterol levels during the 1-year follow-up (Supplementary Fig. S1). Mild proliferation was also noted in both the AAV9-LDLR and AAV9-control groups at both 1 month and 12-month time points, and in the AAV2-control group at the 12-month time point (Fig. 5e and Supplementary Table S1). In one LV-LDLR transduced animal a small, well-defined proliferative area resembling an adenoma was seen (Fig. 5e and Supplementary Fig. S3). No proliferation was seen in the 1-month samples of AAV2-LDLR or AAV2-control groups. It is noteworthy that the proliferative changes in rabbits in all other groups were scored at most as stage 1 (mild) while in the most severe animals in the AAV2-LDLR group the score was 3 (prominent) and the proliferation was seen globally in all liver lobes (Figs 3i,j, 5e and Supplementary Table S1).

### Characterization of the bile duct proliferation

**Figure 6:**
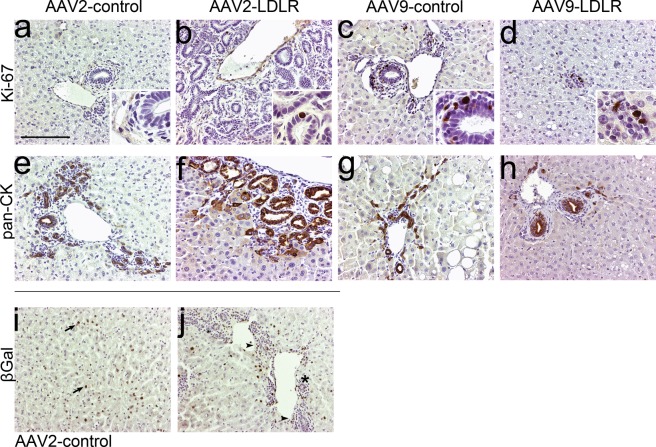
Immunohistochemical characterization of representative rabbit liver samples after AAV mediated gene transfers. (**a–d**) Representative images of Ki-67 staining for proliferating cells in liver sections 1 year after AAV mediated gene transfer of LDLR or control. (**e–h**) Immunohistochemical staining for pan-cytokeratin stained both normal small and large bile ducts (**e,g,h**) and the aberrant tubules in the areas with bile duct proliferation (**f**). (**i,j**) β-galactosidase immunostaining of AAV2-control liver 1 month after gene transfer revealed positive staining in hepatocytes (arrow), inflammatory cells (arrowhead) and biliary epithelium (asterisk). Magnification 200x, inserts 400x. Scale bar: 100 µm.

The bile duct proliferation was assessed as being reactive and not malignant based on the histological features. The aberrant tubules stained with a pan-cytokeratin antibody confirming their epithelial origin (Fig. 6f). Also, histologically normal bile ducts in the AAV2-control and AAV9-control groups, as well as bile ducts in the AAV9-LDLR groups were pan-cytokeratin positive (Fig. 6e,g,h). The antibody also stained hepatocytes around the tubules but almost exclusively in the animals with the most prominent bile duct proliferation (Fig. 6f). Only a few individual pan-cytokeratin positive hepatocytes were seen in AAV2- or AAV9- control animals whereas in the AAV2-LDLR animals pan-cytokeratin positive hepatocytes were seen both lining the bile ducts around the proliferating areas, and also as groups in other parts of the liver tissue (Fig. 6e–h). Pan-cytokeratin positive hepatocytes were not seen in the control livers which had not received any gene transfer (Supplementary Fig. S4).

Immunohistochemical staining with Ki-67 antibody showed some proliferating cells in the areas with bile duct proliferation at the 1-year time point. The frequency was similar to the number of proliferating cells in the normal liver tissue (Fig. 6a–d) and did not differ between the groups. In the 1-month time point the greatest number of proliferating cells was seen in the AAV2-control group where the cells localized mostly in the areas with hematoxylin-stained, small inflammatory cells with some Ki-67 positive cells also in the bile duct epithelium (Supplementary Fig. S5). Only a few individual Ki-67 positive cells were seen in parenchymal cells in any of the groups.

β-galactosidase immunostaining of AAV2-control group liver 1 month after gene transfer showed positive staining in inflammatory cells and biliary epithelium in addition to hepatocytes (Fig. 6i,j).

### Bile duct proliferation is associated with an increased expression of Cyr61 in the liver

**Figure 7:**
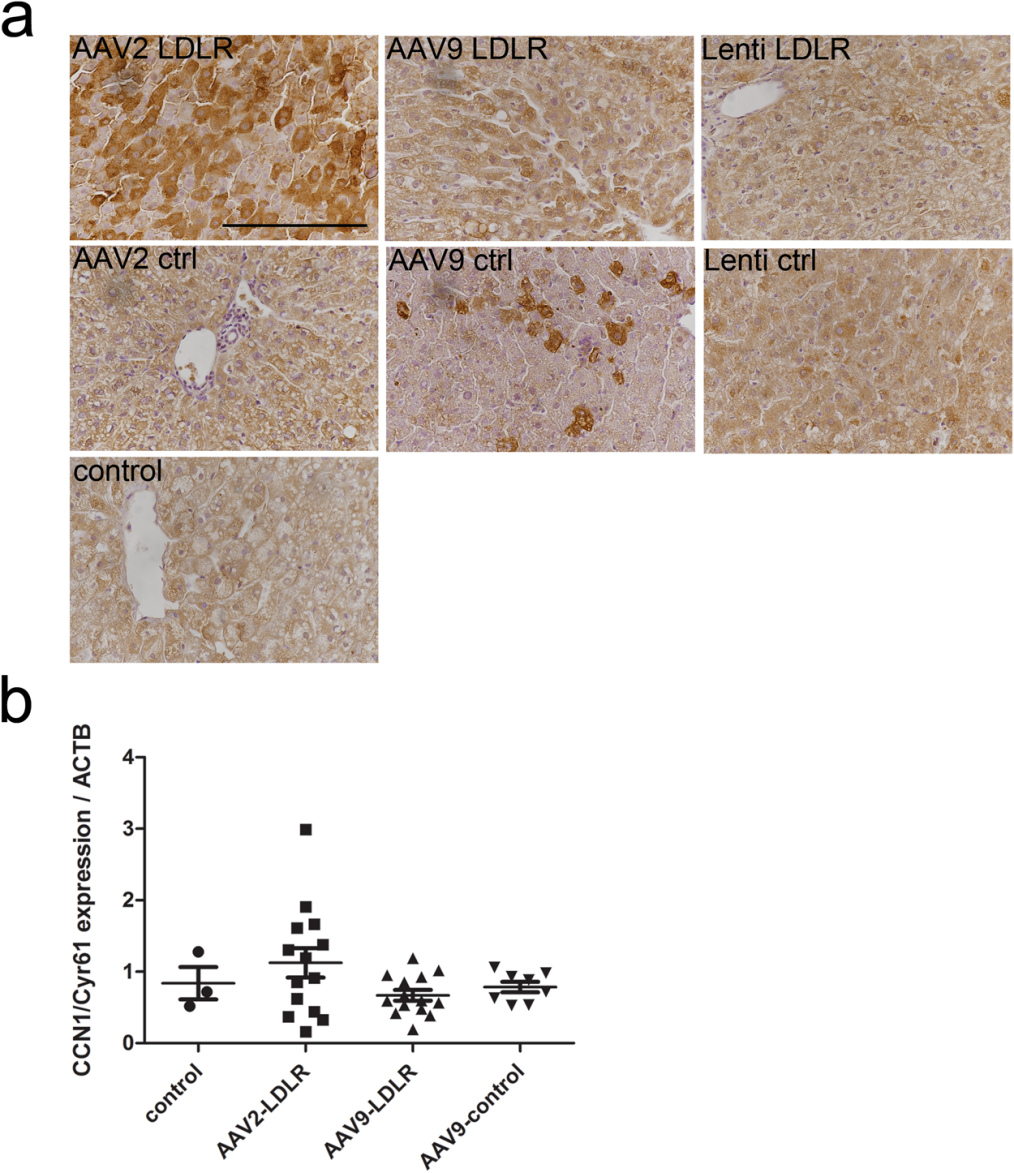
Increased Cyr61 expression in liver after gene transfer with AAV. Immunohistochemistry using anti-rabbit Cyr61 antibody and mRNA expression with RT-qPCR showed increased expression of both protein (**a**) and mRNA (**b**) in livers of AAV2-LDLR transduced rabbits compared to other groups one year after gene transfer. In (**b**) AAV2-LDLR (n = 14; 2–4 liver lobes per animal), control (n = 3), AAV9-LDLR (n = 14, 1–3 liver lobes per animal) and AAV9-control (n = 8, 1–3 liver lobes per animal). Lines in the scatter graph indicate mean ± SEM for each treatment group. Magnification in (**a**) 400x. Scale bar: 100 µm.

Cyr61, a multifunctional matricellular protein, has been shown to ameliorate liver fibrosis and induce cholangiocyte proliferation playing a critical role in ductular reaction. To study the role of Cyr61 in the bile duct proliferation seen after AAV2-LDLR gene transfer, paraffin-embedded sections from rabbits in all AAV and LV transduced groups in addition to sections from naïve, non-transduced rabbit livers were immunostained for Cyr61 expression. It was found that the expression of Cyr61 protein was notably increased in the AAV2-LDLR group compared to the other groups (Fig. 7a). This increase in Cyr61 expression in AAV2-LDLR group was further confirmed with RT-qPCR (Fig. 7b). Notably, the highest expression was seen in the liver samples from the rabbit with the most prominent bile duct proliferation.

### Long-term follow-up

All animals survived gene transfer procedures without complications. One animal receiving AAV2-LDLR died at the 1-month time point due to anesthesia during the liver biopsy. One rabbit receiving AAV9-LDLR vector died 3 months after the gene transfer and another rabbit had to be sacrificed due to paralysis of the hindlimbs after 7 months but these deaths were considered unrelated to the gene transfers.

Analysis of hematoxylin-eosin stained tissues from heart, lung, kidney and spleen of the treated animals revealed some age- and atherosclerosis related changes (Table 1). In most animals from both AAV and LV transduced groups moderate to strong calcification in the tubuli of the kidney was seen (Table 1). Also, modest to strong fibrosis in the kidneys was seen in most animals in both LV-control and LV-LDLR groups. Hemosiderin was noted in the spleens of some animals from all groups (Table 1). No histological changes were seen in the heart in any of the treated groups (Table 1). From one LV-control transduced animal a nephroblastoma was found in the cranial part of the left kidney. However, no provirus could be detected from the nephroblastoma by LAM-PCR analysis (Supplementary Fig. S6).

**Table 1:** Pathological findings in the main non-target tissues.

	Spleen	Lung	Kidney	Heart
AAV2-LDLR	hemosiderin ++	nd	calcification in tubuli +++	n/a
AAV2-control	hemosiderin +	nd	calcification in tubuli +/++	nd
AAV9-LDLR	hemosiderin +/++	nd	calcification in tubuli ++/+++	nd
AAV9-control	nd	lymphocytes +	nd	nd
LV-LDLR	nd	nd	calcification in tubuli +/++; macrophages, cholesterol crystals, connective tissue (fibrosis)	nd
LV-control	nd	nd	calcification in tubuli +/++; macrophages, cholesterol crystals, connective tissue (fibrosis)	nd

Histology of non-target tissues was evaluated from HE-stained sections after the follow-up. Slides were analyzed in a blinded fashion. Changes were scored as nd (not detected), + (mild), ++ (moderate) and +++ (severe) and n/a (not analyzed).

### Expression of LDLR in liver and presence of vector DNA in liver and safety tissues

**Figure 8:**
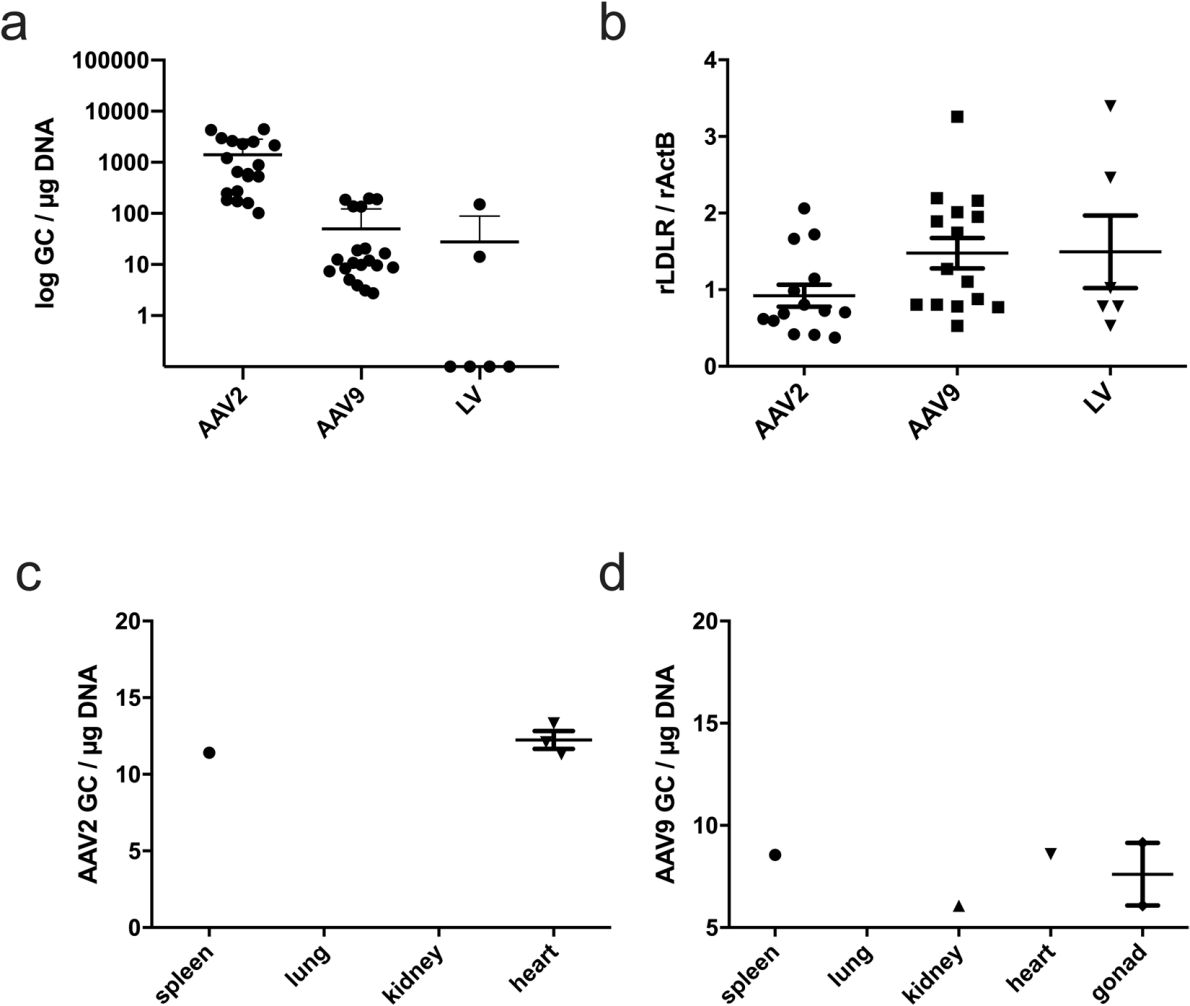
Expression of LDLR in liver and presence of vector DNA in liver and safety tissues after gene transfers. Genome copy numbers in livers 1 year after AAV2-LDLR, AAV9-LDLR and LV-LDLR gene transfers. (**a**) Expression of rabbit LDLR in AAV-LDLR and LV-LDLR transduced livers. (**b**) Very low copy numbers were seen in all biodistribution samples with both AAV2 (**c**) and AAV9 (**d**; n = 3). In (**a,b**) AAV2-LDLR (n = 19; 2–4 liver lobes per animal), AAV9-LDLR (n = 20, 1–3 liver lobes per animal) and LV-LDLR (n = 7). Lines in the scatter graph indicate mean ± SEM.

In the liver samples taken at the time of sacrification 12 months after the gene transfers, vector copy numbers in the liver were similar in AAV9-and LV-LDLR treated groups but were higher in the livers transduced with AAV2-LDLR and AAV9-control (Fig. 8a and Supplementary Fig. S7). Meanwhile mRNA expression levels of rLDLR were similar between all groups after 1 year (Fig. 8b).

Analysis of safety-tissues revealed the presence of vector DNA in the heart, spleen, kidney and gonads from 1–2 animals in very low copy numbers (6–8 GC/µg DNA) in the AAV9-LDLR group (Fig. 8c). For AAV2-LDLR vector copies were detected in spleen and heart samples from 1–3 animals (11–13 GC/µg DNA). From LV-transduced animals provirus was detected in 1/3 of spleen samples (202.7 GC/µg DNA), 2/3 lung samples (15.9 ± 15.7 GC/µg DNA), in 3/3 heart samples (80.3 ± 60.8 GC/µg DNA), in 0/3 kidney samples and in 2/3 gonad samples (2.2 ± 0.3 GC/µg DNA). Generally, copy numbers were higher than in AAV transduced tissues.

## Discussion

In this study we report comparison of preclinical safety, toxicology and efficacy of liver-directed LDLR gene transfers with AAV and LV vectors in WHHL rabbits. Taking into account both efficacy and safety, LV-LDLR vector proved superior to AAV9-LDLR or AAV2-LDLR after long-term follow-up. The initial decrease in total cholesterol was similar in AAV9-LDLR and LV-LDLR rabbits with no statistically significant difference between them. However, due to a decrease in cholesterol levels in AAV9-control group starting six months after the gene transfer the difference between AAV9-LDLR and AAV9-control groups was lost at the 1-year time point. The decline of total cholesterol levels with age is a known phenomenon of the WHHL rabbit colony, and could at least partially explain the decrease seen in the AAV9-control group. AAV2-LDLR gene transfer, on the other hand, was not effective and led to significant pathological changes in liver bile duct histology.

Previously, AAV-mediated gene transfers of LDLR and VLDLR in mouse models of FH have produced significant decreases in serum cholesterol levels and translated also to reductions in the extent of atherosclerosis^11,12,14^. Bissig-Choisat and co-workers demonstrated correction of hypercholesterolemia in a xenograft mouse model of human familial hypercholesterolemia with AAV9 vectors^15^. They showed that while AAV8 and AAV9 were both efficient in transducing hepatocytes, AAV9 was more efficient in transducing human hepatocytes in the xenograft model. Taken together with our findings of a good safety profile of AAV9-LDLR with very few GCs in non-target tissues and the decrease in total cholesterol, at least during the first six months after gene transfer, AAV9 has potential for further development for liver directed gene transfer in humans.

The size of the animal plays a critical role when assessing therapies designed for human use. Partly due to the larger size of WHHL rabbits the viral doses used in this study were lower per kilogram than in the mouse studies but they were still doses that could easily be reached in a human clinical setting. Also, recent questions regarding the safety of high doses of intravenously administered AAV9 and serotype 9-like vectors support using the lowest possible effective titer^16^.

In recently published work Greig *et al*. demonstrated AAV8-LDLR gene transfer into non-human primates in a pre-clinical toxicology study but due to limitations in the animal model in replicating the phenotype of human FH, therapeutic efficacy was not evaluated^17^. The WHHL rabbit has a 12 nucleotide deletion in the *ldlr* gene leading to a defect in receptor internalization. This leads to an increase in serum total cholesterol and LDL, and a human-like lipoprotein profile without high-fat diet and consequently in the development of spontaneous atherosclerotic complications similar to human FH^13^. The relationship between serum cholesterol level and cardiovascular diseases has been convincingly shown in humans and especially in FH. It has been estimated that a 1 mmol/l reduction in plasma LDL cholesterol reduces CAD risk by 20%^18^ so the reduction seen in the LV-LDLR and AAV9-LDLR groups could translate into clinical benefit in homozygous FH patients.

Liver histology was assessed in a blinded manner by a hepatopathologist. Some histological changes, such as the microvesicular- and macrovesicular steatosis seen in the livers of WHHL rabbits can be attributed to severe hypercholesterolemia. In humans steatosis or non-alcholic fatty liver disease (NAFLD) is typically associated with obesity, diabetes and hyperlipidemia^19^. Ballooning degeneration, most commonly associated with steatohepatitis, was noted at low levels in all AAV groups at the early time points. This coincided with the increased numbers of intraepithelial lymphocytes in all groups which might be the cause of the ballooning degeneration. At the end of the study mild ballooning degeneration was seen in a few rabbits in the AAV9-control and LV control groups where the ballooning degeneration was graded as severe. This is most likely due to immune reactions caused by the persistent expression of control genes which are foreign proteins not normally expressed in the rabbits.

Bile-duct proliferation was a new finding after liver-directed gene transfer of 1 × 10^12^ vg of AAV2-LDLR. In the most severe cases the proliferation was seen globally in all liver lobes, and was also clearly visible to the naked eye in the sections. The proliferation was judged as being a reactive, not a malignant change, with the tubules being lined with regular shaped epithelium and normal nuclei. This kind of proliferative change has not been described before after AAV2 gene transfer. Loff and co-workers have described bile duct proliferation related to total parenteral nutrition-induced hepatobiliary dysfunction^20^. The proliferation we saw, however, most closely resembles ductular reaction seen in humans associated with various kinds of liver-injury or diseases, such as drug-induced toxicity or primary biliary cirrhosis^21^. Biliary hyperplasia has also been reported in mice after bile duct ligation^22^. The morphology of the ductular reaction can vary from well-formed ductules with lumina to irregular forms or strings of cells without lumen. Very mild proliferative changes were also noted in individual animals in the AAV2-control and AAV9-control groups whereas the proliferation in the AAV2-LDLR animals was seen globally in all liver lobes. The epithelial cells forming the ducts stained with a pan-CK antibody that also stained normal bile ducts from control rabbits and native controls without any intervention. Staining with the proliferation marker Ki-67 showed a few proliferating cells in the areas with the bile duct proliferation, but the frequency did not differ from the surrounding normal liver tissue or the control animals.

Cyr61 is part of the CCN family of matricellular proteins involved in embryonic development, inflammation and injury repair^23^. The transcription of Cyr61 is rapidly induced by various stimuli, such as growth-factors, inflammatory cytokines and viral and bacterial infections^23^. Recently Cyr61 was shown to play a critical role in the development of ductular reaction and biliary repair after bile duct ligation in the liver of mice by activating cholangiocyte proliferation through integrin α_v_β_5_ –mediated activation of NF-κB^22^. To further investigate the proliferative changes, resembling ductular reaction, in livers of AAV2-LDLR transduced animals we performed immunostaining against Cyr61 and analyzed mRNA expression levels. We saw increased Cyr61 protein expression and mRNA in the livers of AAV2-LDLR rabbits compared to other groups suggesting activation of the Cyr61/α_v_β_5_/NF-κB pathway as a putative mechanism behind the proliferative changes.

Due to its central role in metabolism, high capacity for protein production, large size and good accessibility through the portal vein, the liver has been a widely used target for gene therapy. However, no changes resembling bile duct proliferation have been reported so far even with long follow-up times^17,24^. On the other hand, to our knowledge, no long-term follow-up studies with correction of a metabolic disease have been reported in large animal models.

It might be that increased uptake of LDL in the transduced cells, especially in the bile ducts, persisting for months overwhelm the capacity of the liver to secrete cholesterol into the bile and leads to reactive pathological changes. Although based on our data we cannot claim preferential transduction of cholangiocytes over hepatocytes, the fact that vector copy numbers were higher in AAV2-LDLR transduced livers compared to AAV9-LDLR while the decrease in serum cholesterol was greater in the AAV9-LDLR livers may suggest transduction of other cell types. These cells might not able to process increased intracellular LDL thus disturbing normal cellular function.

Indeed, proliferation of cholangiocytes is known to occur in response to many pathological conditions in the liver^25^. We saw increased numbers of intraepithelial lymphocytes in the AAV2-groups at the 1-month time point compared to AAV9-groups and lymphocytes were seen in the portal fields of AAV2-transduced animals after one-year follow-up. Cyr61 expression has been shown to induce hepatic inflammation with increased infiltration of macrophages via integrin α_M_β_2_ in mouse models of NAFLD. Increased expression of Cyr61 is also caused by endotoxins and FFAs in hepatocytes^26^.

Kassim and co-workers showed a significant decrease in plasma total cholesterol levels after AAV8-LDLR gene transfer into *ldlr*^−/−^/*apobec*^−/−^ mice, and did not report any pathological changes in the liver^12^. However, the lack of pathological changes was determined by the analysis of hepatic cholesterol and triglyceride content from homogenized tissue and liver histology was not reported. No liver pathology has been reported in long-term, liver-directed gene transfer of factor IX by AAV vectors for haemophilia B in canines^27^ or intra-arterial AAV8-LDLR gene transfer into non-human primates^17^. Normal liver function tests including ALT, AST and bilirubin in our study indicated, however, that despite the bile duct proliferation overall function of the liver was not impaired.

In summary, LV mediated gene transfer was more efficient than AAV2 or AAV9 in lowering serum cholesterol levels in hypercholesterolemic WHHL rabbits. Significant bile duct proliferation was seen in AAV2-treated animals and it was likely caused by an increased expression of Cyr61 in the liver. In light of our results further long-term safety studies with AAV-vectors are required to elucidate their efficiency and possible long-term effects in the liver. Of the currently tested vectors lentiviral LDLR construct could be brought to human clinical testing in homozygous FH patients.

## Methods

### Production of viral vectors

AAV2 and AAV9 LDLR vector contructs contained identical expression cassettes of rabbit LDLR with the synthetic liver-specific α1-microglobulin/bikunin (ABP)/thyroid hormone-binding globulin (TBG) enhancer/promoter (LSP) ABP/TBG promoter^7^ and LacZ was used as a control gene.

AAV2 vectors were produced as previously described^28^ with some modifications. Briefly, 293 T cells were transfected with AAV2 vector plasmid and pDG helper plasmid (kindly provided by Dr Jurgen Kleinschmidt, DKFZ, Heidelberg, Germany) with calcium phosphate precipitation. Fresh medium was changed after 24 h and cells were harvested 48–72 h after transfection. Viral vector was released from cells by 3 freeze-thaw cycles. The vector-containing media was then purified by iodixanol-gradient centrifugation and heparin-affinity chromatography. Fractions containing the purified vector were collected and dialysed against PBS. The purified vector was stored in PBS at −70 °C until use.

AAV9 vectors were prepared at the Vector Development Core Lab at UCSD as previously described^29^. Helper virus-free AAV9 vectors were produced by transient transfection of HEK293T cells with the vector plasmid, pRep2-Cap9 and pAd-Helper plasmid^30^. Plasmid pRep2-Cap9 plasmid was obtained from Dr. Wilson (U. Penn). Cell lysates prepared at 72 h after transfection were treated with benzonase and viruses were pelleted through 25% sucrose-cushion ultracentrifugation. The pellets were resuspended and the viruses were further purified through anion-exchange column chromatography (Q-Sepharose, GE Health Science)^31,32^ followed by concentration through 25% sucrose-cushion ultracentrifugation. The final pellets were resuspended in 10 mM Tris-HCl, pH7.9, 1 mM MgCl2, 3% sucrose. Virus titers were determine ed by measuring the genome copies by Real-time Q-PCR using virus genome DNAs prepared from the purified virus preparations.

LV vectors encoding rabbit LDLR under the transcriptional control of the same ABP/TBG promoter^33^ were produced in 293 T cells as previously described^7^ and GFP was used as a control gene.

### *In vivo* gene transfer

WHHL rabbits (n = 54, male and female; 2.5–3.5 kg) were used for *in vivo* gene transfer of AAVs. AAV2 and AAV9 serotypes were used for portal vein injections into WWHL rabbit liver. A total of 1 × 10^11^–1 × 10^12^ vg of AAV vectors diluted in 2–5 ml 0.9% NaCl were injected per animal of each of the following vectors: AAV2-LDLR (n = 8), AAV2-control (n = 11), AAV9-LDLR (n = 15) and AAV9-control (n = 10). For LV vectors 1 × 10^9^ IU (10 µg of p24 gag-antigen) of LV-LDLR (n = 6) and LV-control (n = 4) were used^7^.

Briefly, animals were ansthesized with ketamine (Ketalar 20 mg/kg, Pfizer) and medetomidine (Domitor 0.3 mg/kg, Orion, Finland). Laparotomy was performed, and the portal vein visualized. Vectors were injected via a 24G cannula into the portal circulation. After removal of the cannula the puncture site was observed to make sure no bleeding occurred after which the laparotomy wound was closed in two layers. Carprofen (Rimadyl 4 mg/kg, Pfizer) was given as a post-operative analgesic. Animals were sacrificed 1 month or 1 year after gene transfer.

### Clinical chemistry

Alanine aminotransferase (ALT), alkaline phosphatase (APT), aspartate aminotransferase, total cholesterol (tot chol), low-density lipoprotein (LDL), high-density lipoprotein (HDL) and triglycerides (trigly) were monitored from fasting serum samples regularly. Samples were collected at baseline and monthly thereafter for the duration of the study and analyzed at MoVet veterinary service laboratory (MoVet, Kuopio, Finland).

### Histology

Tissue samples for histological analysis were taken at the time of sacrifice from liver, spleen, skeletal muscle, heart, gonads, lung and kidney. In order to ensure that all the liver was accurately represented, samples from four liver lobes per animal were collected. Samples were fixed in 4% PFA-sucrose, embedded in paraffin and 7 µm sections were cut. Sections were stained with hematoxylin-eosin (HE) for analysis of liver histology. Histology was assessed in a blinded manner by a hepatopathologist (V.K.) from 4–5 slides/liver and changes were graded according to severity and/or prominence of each change from 0 to 3^34^. The average score for each treatment group ± SEM is shown.

Immunohistochemical staining was done for macrophages (1:200, RAM-11; DAKO), proliferating cells (1:200, Ki-67; DakoCytomation), rabbit T-lymphocytes (1:200; GenWay), Cyr61 (5 µg/ml; Cyr61, Novus Biologicals;), β-galactosidase (1:200; β-gal; Promega) and cytokeratins (1:200, pan-Cytokeratin, AE1/AE3; Santa Cruz Biotechnology). Photomicrographs of the 7 µm histological sections were taken with an Olympus AX70 microscope (Olympus Optical) and analySIS software (Soft Imaging System). Images were further processed for publication with Adobe Photoshop 7.0 (Adobe).

### qPCR and RT-qPCR

DNA was extracted from snap-frozen tissue samples liver after Proteinase K treatment and extracted with phenol-chloroform according to standard protocols. For LV-LDLR tissues DNA was isolated from formalin-fixed paraffin embedded (FFPE) tissue as previously published^35^. Ten sections of 10 µm each from each FFPE specimen were used for isolation of gDNA. PCR amplification was carried out on 50 ng or 500 ng (FFPE-samples) of genomic DNA. Vector copy numbers were analysed using TaqMan custom assay for LSP promoter (Applied Biosystems; forward primer 5′-GCCTCTGCTTTTGTACAACTTTCC-3′, reverse primer 5′-AGTTCTCACTATTGGGCCAAACAG-3′ and probe AAAACTGCCAATCCC) or PrimePCR™ custom Probe assay for WPRE (Bio-Rad; forward primer 5′-ATACGCTGCTTTAATGCCTTTG-3′, reverse primer 5′-GGGCCACAACTCCTCATAAA-3′ and probe TCATGCTATTGCTTCCCGTATGGCT) and 2x Universal Master Mix. Real-time qPCR was carried out in an ABI Step One Plus Prism 7700 and data was analysed with the corresponding software (Applied Biosystems). The detection limit for the custom assays was determined with plasmid DNA of known target copy numbers spiked with 50 ng of genomic DNA and was determined as 10 copies. RNA was isolated from snap-frozen tissue samples with TRI-reagent (Sigma Aldrich) according to the manufacturer’s instructions. A total of 1 µg of RNA was used for cDNA synthesis after DNAse treatment (Turbo DNA free RNA kit; Thermo Fischer Scientific) using random hexamer primers (Promega) and Revert Aid™ (Thermo Fischer Scientific). The relative expression levels of Cyr61 and rLDLR in liver samples from the different treatment groups were measured according to the manufacturers’ protocol using either rabbit Cyr61 PrimePCR™ Probe Assay (Bio-Rad; assay ID: qOcuCEP0031536) or PrimeTime custom qPCR Probe assay for rabbit LDLR (IDT DNA; forward primer 5′-GTATCTCCTACAAGTGGGTGTG-3′, reverse primer 5′-GACTTGCAGGTGAGA GACAT-3′and probe/56-FAM/CG GCT CGG A/Zen/C GAG TGG GAG CAG/3IABkFQ/) with TaqMan universal 2x PCR mix (Life Technology). The expression levels were normalized to rabbit ActB (IDT DNA; forward primer 5′-GGACCTGACCGACTACCT-3′, reverse primer 5′-GTAGCACAGCTTCTCCTTGAT-3′ and probe/56-FAM/ATGAAGATC/Zen/CTCACGGAGCGCG/3IABkFQ/).

### Statistical analysis

The results are expressed as mean ± SEM. Statistical significance was analyzed by one-way ANOVA followed by Tukeys multiple comparison test or Student’s t-test where appropriate (GraphPad Prism). Clinical chemistry data was analysed with linear mixed-effects model using the R Statistical Software by looking at the effects of group, treatment and time on the response (Foundation for Statistical Computing, Vienna, Austria, version 3.2.5).

The model for rabbit *i* at time point j was of form,

$${y}_{ij={x}_{ij}^{\text{'}}}\beta +{u}_{i}+{e}_{ij}$$

where u_i_ is normally distributed random effects with zero mean and constant variance and e_ij_ is a zero-mean residual. In the case of non-constant variance the variance of residual errors were modelled with a proper variance function and co-variances between error term for a particular rabbit were modelled using AR1 or AR2 process if necessary.

The fixed part includes the levels of group (AAV2, AAV9 or LV) or two levels of treatment (LDLR and control) and function of time. In the model the effect of time was modelled through natural splines with four knots at time points 3, 5, 7 and 10^36^. The interactions between the spline function and group or treatment were also included in the models. A p-value below 0.05 was considered statistically significant.

### Study approval

All animal experiments were approved by the Animal Experiment Board in Finland and carried out in accordance with The Finnish Act on Animal Experimentation. The investigation conforms with the *Guide for The Care and Use of Laboratory Animals* published by the US National Institutes of Health (NIH Publication No. 85-23, revised 1996).

## Supplementary information

Supplementary information

## Data Availability

All data generated or analysed during this study are included in this published article, and its supplementary dataset.
