# Supplementary material for: Bile-duct proliferation as an unexpected side-effect after AAV2-LDLR gene transfer to rabbit liver
Source: Sci Rep. 2019 May 6;9:6934. doi: 10.1038/s41598-019-43459-1 (PMC6502883; doi:10.1038/s41598-019-43459-1)
Supplement: Supplementary file 1 — Supplementary information [file 41598_2019_43459_MOESM1_ESM.pdf]

Supplementary information for

Bile-duct proliferation as an unexpected side-effect after AAV2-LDLR gene transfer to rabbit liver

Hytönen Elisa[1], Laurema Anniina[1], Kankkonen Hanna[2], Miyanohara Atsushi[3], Kärjä Vesa[4], Hujo Mika[5], Laham-Karam Nihay[1] and Ylä-Herttuala Seppo [1,6,7] *

[1] A. I. Virtanen Institute for Molecular Sciences and Department of Medicine, University of Eastern Finland, Neulaniementie 2, FIN-70210 Kuopio, Finland.

[2] BioMediTech Institute and Faculty of Medicine and Life Sciences, University of Tampere, Tampere, Finland

[3] Department of Pediatrics, UC San Diego School of Medicine, La Jolla, CA, USA.

[4] Department of Pathology, University of Eastern Finland, Kuopio, Finland

[5] School of Computing, University of Eastern Finland, 70211 Kuopio, Finland

[6] Heart Center, Kuopio University Hospital and [7)] Gene Therapy Unit, Kuopio University Hospital, FIN-70210 Kuopio, Finland

[*] Corresponding author:

Seppo Ylä-Herttuala, M.D., Ph.D. FESC; Professor of Molecular Medicine

A. I. Virtanen Institute for Molecular Sciences

University of Eastern Finland, P. O. Box 1627

FIN-70211 KUOPIO, FINLAND

Tel. +358-40-355 2075

Fax +358-17-163 751

E-mail: Seppo.Ylaherttuala@uef.fi

Supplementary Table S1. Pathological changes in liver 1 and 12 months after gene transfer

Change in liver	AAV2-LDLR		AAV2-control		AAV9-LDLR		AAV9-control		lenti-LDLR	lenti-control
	1 mo	12mo	1mo	12mo	1mo	12mo	1mo	12mo	12mo	12mo
Bile duct proliferation	−	+ +	−	+	+	−	+	+	−	−
Macrovesicular steatosis	+	+ +	+ +	+	+ +	+ +	+ +	+ +	−	+
Microvesicular steatosis	+ +	+ +	+ +	+ + +	+ +	+ +	+ +	+ + +	−	+
Stasis	+	+	+	+	+	+	−	+	−	−
Lymphocytes in portal fields	−	+	−	+	−	−	−	−	−	−
Ballooning degeneration	+	−	+ +	−	+ +	−	+ +	+	−	+ + +
Inclusions	+	−	+	−	−	−	−	+	−	+
Bile-duct intraepithelial lymphocytes	+ +	+	+ +	+	+	+	+	+	+	+
Eosinophilic granulocytes in portal fields	−	+	−	+	+	+	+	+	−	−
Interphase inflammation	−	−	−	−	−	−	−	−	−	−

Liver histology was evaluated from hematoxylin and eosin stained sections. Slides were scored on a semi-quantitative scale by a pathologist (V.K.) blinded to the origin of the samples. Four slides from each individual animal were scored twice, the average/animal was calculated and from these the average for each treatment group was calculated. – no change; + mild change; ++ moderate change and +++ abundant change.

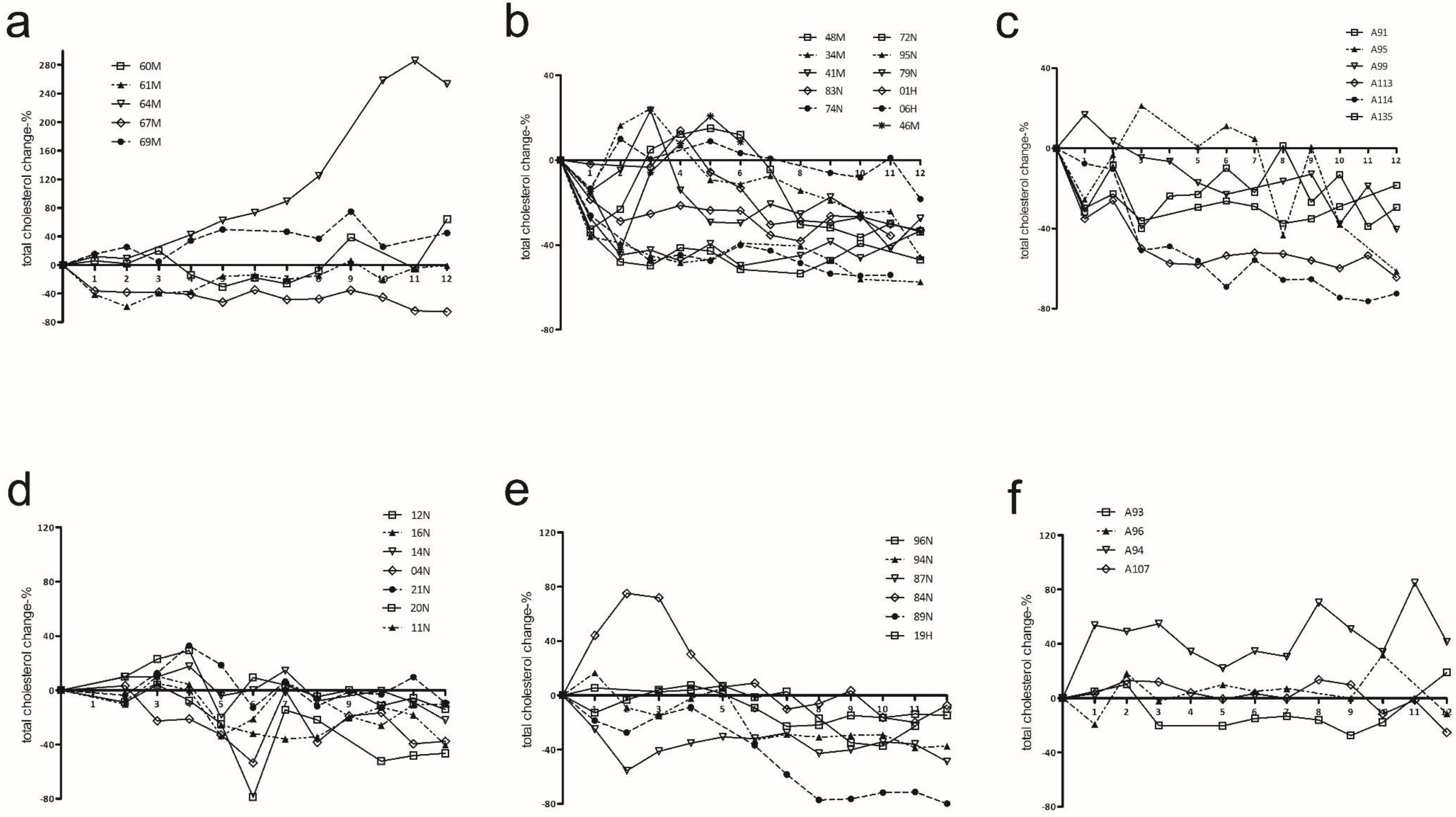

a
60M
61M
64M
67M
69M
total cholesterol change-%
b
48M
34M
41M
83N
74N
72N
95N
79N
01H
06H
46M
total cholesterol change-%
c
A91
A95
A99
A113
A114
A135
total cholesterol change-%
d
12N
16N
14N
04N
21N
20N
11N
total cholesterol change-%
e
96N
94N
87N
84N
89N
19H
total cholesterol change-%
f
A93
A96
A94
A107
total cholesterol change-%

Supplementary Figure S1. Long-term follow-up of total cholesterol values of individual animals after intraportal gene transfer. Total cholesterol, represented as %-change from pre-treatment values, for each animal is shown over a 1-year period (1-12 months). Figure S1 (a) represents AAV2-LDLR, (b) AAV9-LDLR, (c) lenti-LDLR, (d) AAV2-control, (e) AAV9-control and (f) lenti-control.

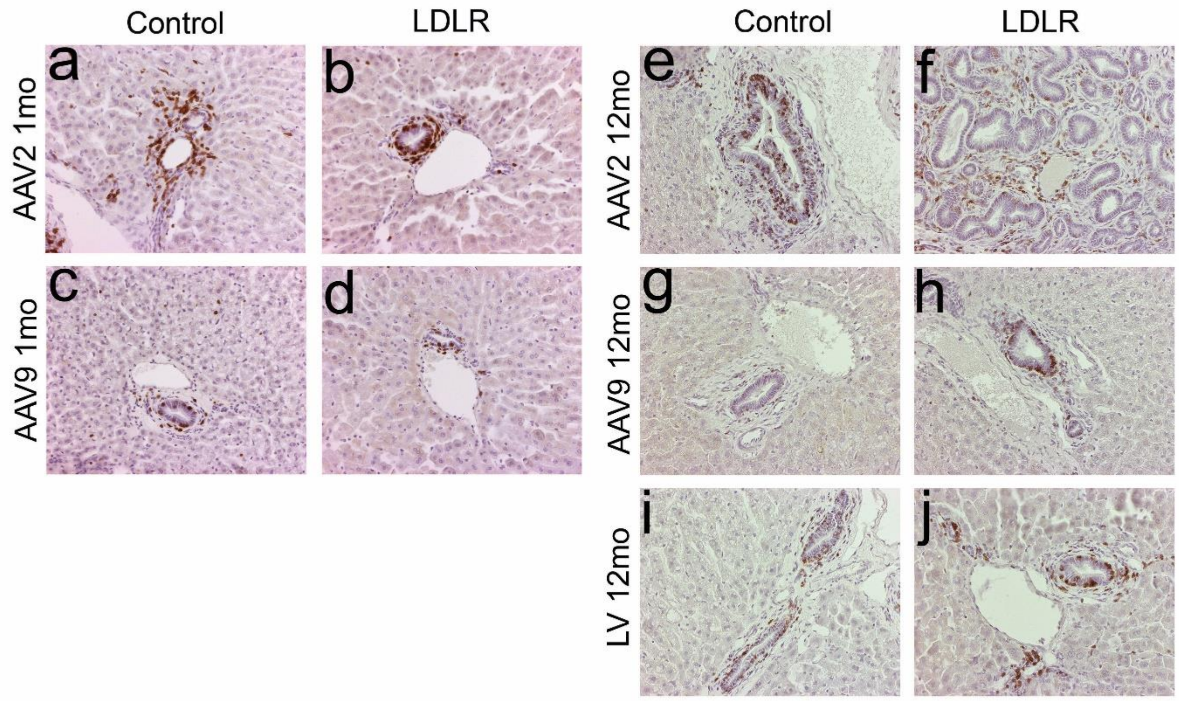

Supplementary figure S2. Immunohistochemical staining for lymphocytes in representative liver samples one month and one year after gene transfer. Bile duct intraepithelial lymphocytes (IEL) and lymphocytes in portal fields were identified with immunostaining with an antibody against rabbit T-lymphocytes. IELs were most prominent in AAV2 transduced rabbits one month after gene transfer (a and b) whereas the levels of IELs were similar in all groups at the end of the follow-up (e-j). Magnification 200x.

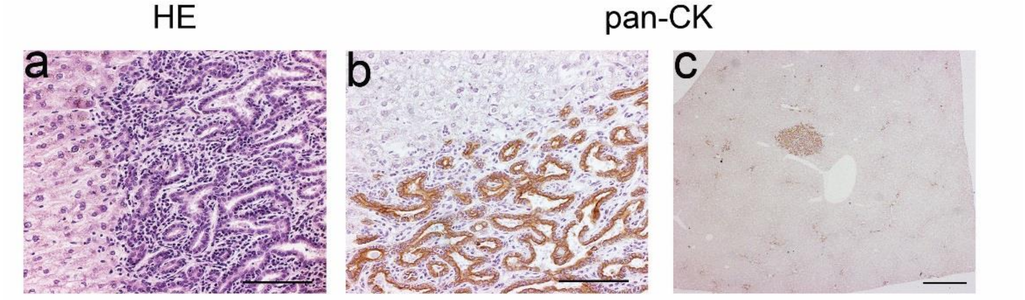

Supplementary Figure S3. Characterization of adenoma in lenti-control transduced WHHL rabbit. HE-staining (a) and pan-CK immunohistochemistry (b and c) show tubular structure of single adenoma found in one lenti-control transduced animal. Magnification (a, b) 200X, scale bar 100 µm and (c) 12.5x, scale bar 1000 µm.

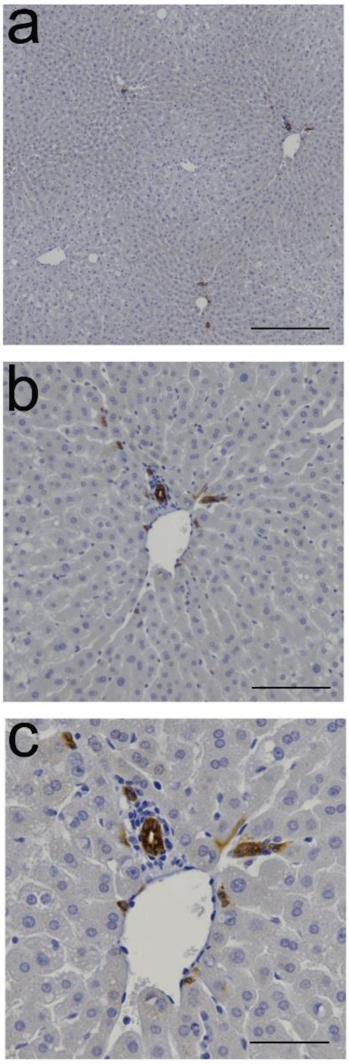

Supplementary Figure S4. Immunohistochemical staining for pan-cytokeratin in normal rabbit liver. Immunohistochemical staining of untreated WHHL rabbit liver with pan-cytokeratin antibody showed positive staining of bile ducts with no staining of hepatocytes (a-c). Magnification (a) 40x, scale bar 500µm; (b) 100x, scale bar 200µm and (c) 200x, scale bar 100µm.

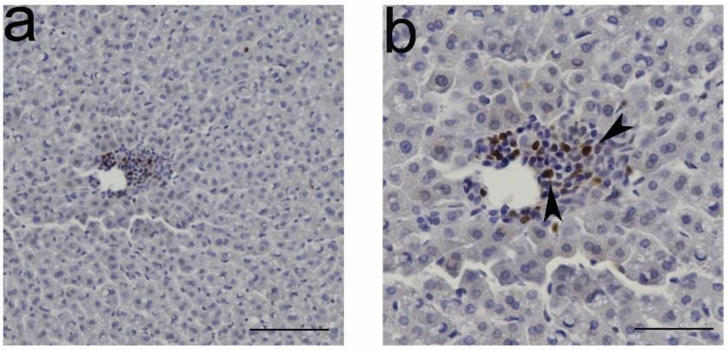

Supplementary Figure S5. Immunohistochemical staining for proliferating cells. Proliferating cells were seen in AAV2-control rabbit liver with Ki-67 staining localizing in areas of inflammatory cells (arrowheads). Magnification (a) 100x, scale bar 200µm and (b) 200x, scale bar 100µm.

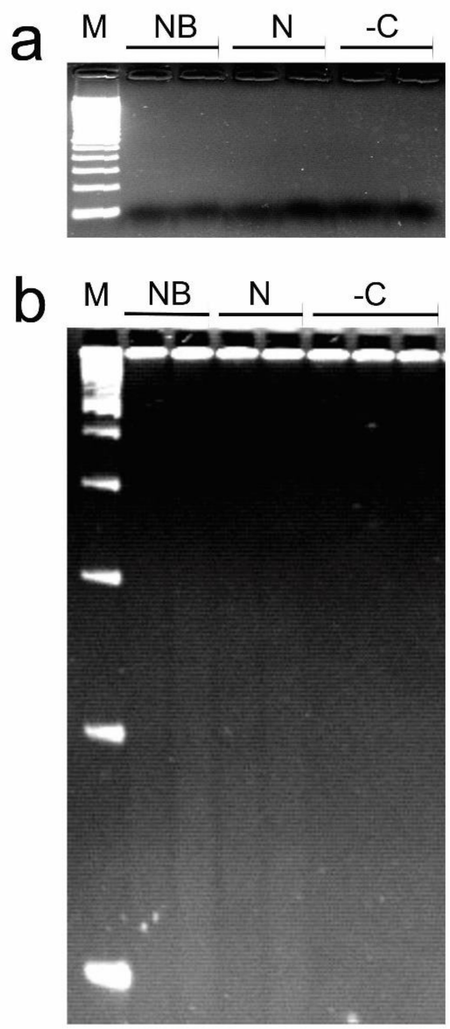

Supplementary Figure S6. LAM-PCR analysis for provirus integration in nephroblastoma of the kidney. No provirus could be detected by vector-LTR nested PCR (a) or linear-amplification mediated PCR (b) from a nephroblastoma tumour found in a LV-control transduced rabbit. M: 100bp ladder, NB: nephroblastoma, N: normal tissue section, -C: H_2O control.

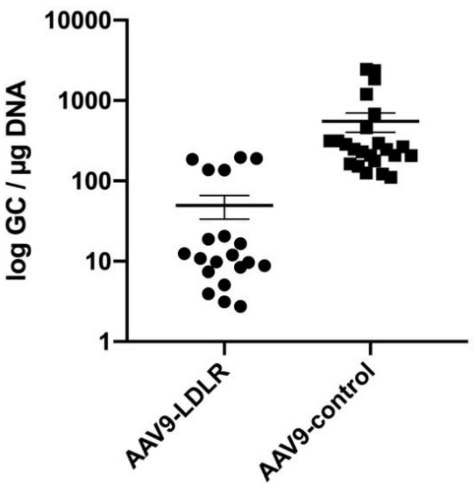

Supplementary Figure S7. Copy numbers in liver one year after AAV9-rLDLR and AAV9-control gene transfers. PCRs were run 2-3 times for each animal. AAV9-LDLR (n=20, 1-3 liver lobes per animal) and AAV9-control (n=23, 1-3 liver lobes per animal). Lines in the scatter graph indicate mean ± SEM.
